# Lipomatous Hypertrophy of the Atrial Septum in a Patient Undergoing Coronary Artery Bypass Surgery

**DOI:** 10.1155/2016/2080875

**Published:** 2016-12-19

**Authors:** Thomas Strecker, Michael Weyand, Abbas Agaimy

**Affiliations:** ^1^Center of Cardiac Surgery, Friedrich-Alexander University, Erlangen, Germany; ^2^Institute of Pathology, Friedrich-Alexander University, Erlangen, Germany

## Abstract

*Background*. Lipomatous hypertrophy of the atrial septum (LHAS) is a rare entity characterized by mass-forming deposition of fatty tissue within the atrial septum. To date, <300 cases have been reported; many of them were autopsy findings. The clinical presentation of LHAS varies from incidental asymptomatic mass (most frequent form) to severe life-threatening cardiovascular complications necessitating emergency cardiac surgery.* Case Presentation*. Here, we present the successful surgical resection of such a massive LHAS which was found incidentally on preoperative investigation of a 71-year-old patient with progressive coronary heart disease. Histology confirmed the diagnosis of lipomatous hypertrophy of the atrial septum.* Conclusions*. The described case report illustrates an unusual example of LHAS in a patient undergoing a planned coronary artery bypass surgery. In this case, surgical intervention was justified to avoid later outflow obstructions.

## 1. Introduction

Lipomatous hypertrophy of the atrial septum (LHAS) is a relatively uncommon benign abnormality characterized by accumulation of unencapsulated mass-forming mature fat within the atrial septum [1, 2]. The reported incidence of LHAS in the general population varied between 1% and 2% [3, 4]. Defined as atrial septum thickening >2 cm, this lesion is frequently confused with other cardiac tumors [5, 6]. LHAS could be associated with congestive heart failure and various cardiac arrhythmias, such as atrial fibrillation or supraventricular tachycardia [7].

The radiological evaluation of cardiac mass-forming lesions has been greatly facilitated by the development of high-resolution noninvasive cardiac imaging [8, 9]. Nevertheless, transthoracic as well as transesophageal echocardiography is often not specific enough to establish diagnosis of LHAS [10, 11]. Echocardiographic guidelines for the diagnosis of LHAS were described by Fyke and colleagues [12]. In some of the cases, LHAS requires surgical excision to prevent severe cardiovascular complications.

This article demonstrates the necessity to also include LHAS in the differential diagnosis of subtle and nonspecific cardiothoracic symptoms.

## 2. Case Presentation

A 70-year-old Caucasian male with coronary heart disease and symptoms of heart failure was referred to our hospital. He noted progressive dyspnea, arterial hypertension, and exhaustion. At admission, the patient presented a blood pressure of 150/90 mmHg and a heart rate of 80 beats per minute. Transthoracic echocardiography (TTE) revealed a dilated left atrium and ventricle with a highly impaired left ventricular ejection fraction of 30%. The heart valves appeared almost unremarkable with slight aortic and mitral valve regurgitation. Subsequent invasive coronary angiography showed significant stenosis of the ramus circumflex (RCX) and the right coronary artery (RCA); the left main and the anterior descending artery showed slight narrowing only.

The patient was taken to the operating theatre, where a median sternotomy was performed and cardiopulmonary bypass was performed through aorto-right atrium cannulation. Intraoperative transesophageal echocardiography (TEE) demonstrated a large mass within the interatrial septum. After opening of the right atrium (RA), the large retractile sessile mass was successfully excised. The resulting defect in the RA was then closed with a pericardial patch. Afterwards, two single saphenous vein grafts were anastomosed to the RCX and the RCA. The patient was weaned from cardiopulmonary bypass without any signs of cardiac failure. He made an uneventful recovery, was extubated on postoperative day 2, and transferred to the intermediate care on day 4. Before leaving the hospital on day 9 after surgery, the TTE showed a moderate impaired ventricular function, well-functioning heart valves, and no pericardial effusions.

## 3. Results

### 3.1. Pathological Features

**Figure 1:**
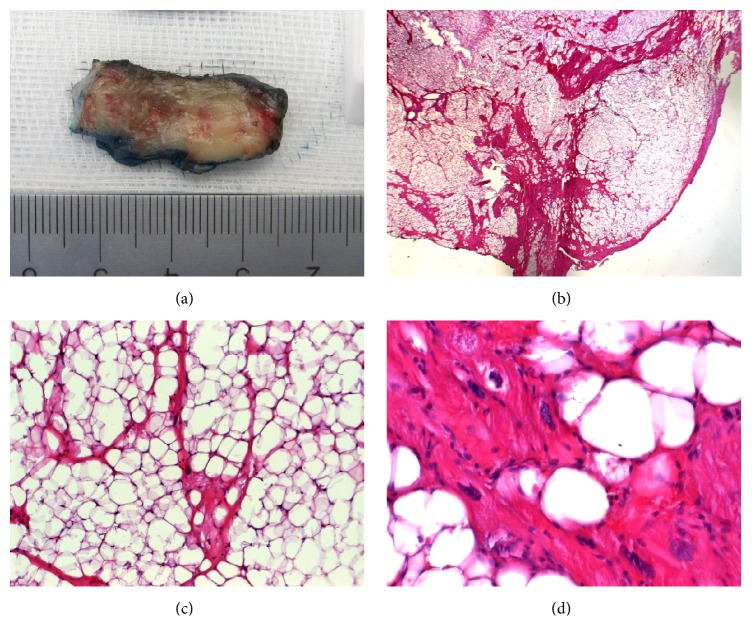
(a) Gross feature of LHIS showed diffuse thickening of the interatrial septum with yellow cut surface. (b) Histological overview showed extensive fat entrapping cardiomyocytes. (c) High power shows mature adipocytes and residual muscle forming septa. (d) High power of myocytes shows enlarged bizarre nuclei mimicking liposarcoma.

The gross specimen measured 3,7 × 2,5 × 1,2 cm and was covered by glistening endocardium. On cut surface, it was entirely composed of yellowish smooth glistening fatty tissue occupying and expanding the atrial septum (Figure 1(a)). Histological examination showed extensive nodular thickening of the interatrial septum due to excessive accumulation of mature adipose tissue which reached the resection surface (Figure 1(b)). At higher magnification, the adipocytes were mature without any cellular or nuclear atypia (Figure 1(c)). A few brown fat cells were seen as well occasionally mimicking lipoblasts. The fatty tissue was dissected by residual muscle fibers with variable reactive nuclear enlargement occasionally mimicking atypical stromal cells of liposarcomas (Figure 1(d)). However, these cells were unequivocally recognizable as cardiomyocytes by their cytoplasmic characteristics. There were no true lipoblasts, atypical stromal cells, or mitotic figures. These finding were consistent with LHAS.

## 4. Discussion

Benign lipomatous tumors of the heart are exceptionally rare. They encompass mainly lipoma and LHAS. In a recent large series of 47 cases collected over a period of 19 years at the Mayo Clinic [13], only five lipomas were encountered. However, of the 42 cases of LHAS retrieved in that study, 39 lesions were autopsy findings and only 3 cases have been resected surgically thus highlighting the rarity of clinically diagnosed and resected LHAS.

LHAS is rare [14, 15]. Since its first description in a series of five autopsy cases by Pior in 1964 [16], less than 300 cases have been reported [13, 17]. Although many histogenetic theories have been suggested, the etiology of LHAS is still unknown [18]. However, the popular theory relating LHAS to proliferation of mesenchymal/fatty tissue entrapped during embryological life has been recently challenged by demonstration of* HGMA2* gene rearrangement in one case of LHAS suggesting a true neoplasm, but the limited number of cases examined does not allow for definitive conclusion regarding this point. There are no typical clinical symptoms of LHAS, but atrial arrhythmias, like atrial fibrillation and supraventricular tachycardia, are common [7]. Usually LHAS remains asymptomatic and represents an incidental finding on echocardiography. Furthermore, it can be mistaken for other benign or malignant cardiac tumors leading to unwarranted radical surgical resection [19, 20].

The diagnosis of LHAS is made either preoperatively with noninvasive imaging techniques or coincidentally at time of cardiac surgery like in the present case. The usual imaging to evaluate LHAS is transthoracic and transesophageal echocardiography, where the septum appears unusually thick sparing the membrane of the* fossa ovalis* [19, 21]. Surgical resection of LHAS is essentially not necessary and should be reserved for patients who show evidence of right or left outflow obstruction or vena cava superior obstruction [22, 23]. Histologically, LHAS is distinguished from lipoma by encapsulation of the latter and from rare cardiac liposarcomas by absence of atypical lipoblasts, atypical stromal cells, and other features of malignancy. The presence of reactive nuclear enlargement of entrapped cardiomyocytes might be mistaken for the rare case of cardiac liposarcoma. However, these cells are readily recognizable as cardiac muscle cells by their characteristic cytoplasmic features including prominent eosinophilia and cross-striation. In equivocal cases, MDM2 and CDK4 assessment by immunohistochemistry and/or molecular techniques (FISH) are helpful to exclude liposarcoma.

## 5. Conclusions

The reported case illustrates an unusual example of LHAS in a patient undergoing a planned coronary artery bypass surgery. In these cases, surgical intervention is justified to avoid later outflow obstructions. The histological examination of such cardiac lesions is mandatory to exclude other fatty masses of the heart, in particular liposarcomas.

## Abbreviations

LHAS: Lipomatous hypertrophy of the atrial septum
TEE: Transesophageal echocardiography
TTE: Transthoracic echocardiography
LAD: Left anterior descending artery
RCX: Ramus circumflex
RCA: Right coronary artery
RA: Right atrium.
